# Androgen Receptor-Dependent and -Independent Mechanisms Involved in Prostate Cancer Therapy Resistance

**DOI:** 10.3390/cancers9060067

**Published:** 2017-06-12

**Authors:** Daniel J. Crona, Young E. Whang

**Affiliations:** 1 Division of Pharmacotherapy and Experimental Therapeutics, Eshelman School of Pharmacy, University of North Carolina, Chapel Hill, NC 27599, USA; crona@email.unc.edu; 2 Lineberger Comprehensive Cancer Center, University of North Carolina, Chapel Hill, NC 27599, USA; 3 Division of Hematology and Oncology, Department of Medicine, School of Medicine, University of North Carolina, Chapel Hill, NC 27599, USA

**Keywords:** castration-resistant prostate cancer, androgen receptor, progression, resistance mechanisms, enzalutamide, abiraterone

## Abstract

Despite the initial efficacy of androgen deprivation in prostate cancer, virtually all patients progress to castration-resistant prostate cancer (CRPC). Androgen receptor (AR) signaling is critically required for CRPC. A new generation of medications targeting AR, such as abiraterone and enzalutamide, has improved survival of metastatic CRPC (mCRPC) patients. However, a significant proportion of patients presents with primary resistance to these agents, and in the remainder, secondary resistance will invariably develop, which makes mCRPC the lethal form of the disease. Mechanisms underlying progression to mCRPC and treatment resistance are extremely complex. AR-dependent resistance mechanisms include *AR* amplification, *AR* point mutations, expression of constitutively active *AR* splice variants, and altered intratumoral androgen biosynthesis. AR-independent resistance mechanisms include glucocorticoid receptor activation, immune-mediated resistance, and neuroendocrine differentiation. The development of novel agents, such as seviteronel, apalutamide, and EPI-001/EPI-506, as well as the identification and validation of novel predictive biomarkers of resistance, may lead to improved therapeutics for mCRPC patients.

## 1. Introduction

In the United States, prostate cancer is the most commonly diagnosed malignancy (aside from skin cancer), where approximately one out of every seven men will be diagnosed with the disease during their lifetime [1]. It is estimated that 161,360 new cases of prostate cancer will be diagnosed in 2017 [1,2]. Overall survival (OS) for prostate cancer has improved over the past four decades, likely due to a combination of early detection and diagnoses with improved treatment options. The 5-year survival rate for prostate cancer (all stages combined) has risen from 68% to 99% [1,3]. The 10-year survival rate is 98%, while the 15-year survival rate is 95% [3].

However, prostate cancer is still the third leading cause of cancer-related death among men in the US. It is estimated that 26,730 deaths will occur in 2017 due to prostate cancer [2]. Despite surgery or radiation, some patients (perhaps up to 20–30%) with clinically localized prostate cancer will have recurrence of their disease after treatment and will progress to the metastatic stage over time. For metastatic prostate cancer, androgen deprivation therapy (ADT) is the standard treatment, and ADT can be achieved either by surgical castration through bilateral orchiectomy or medical castration through the use of luteinizing hormone-releasing hormone (LH-RH) agonists (i.e., leuprolide acetate) or LH-RH antagonists (i.e., degarelix acetate). Despite its initial effectiveness in stabilizing or causing regression of metastatic prostate cancer, progression to the lethal form of the disease, known as castration-resistant prostate cancer (CRPC), is essentially inevitable for these patients. CRPC can be defined as either progressively rising levels of serum tumor marker prostate-specific antigen (PSA) or detection of new or progressive metastatic tumors by radiographic scans, despite castrate testosterone levels (≤50 ng/dL). Based on recent preclinical and clinical data, it is now evident that CRPC is not “androgen-independent” despite systemic depletion of androgens, but rather continues to be dependent on the androgen receptor (AR) signaling axis [4,5]. Two new agents, abiraterone and enzalutamide, have been recently approved by the U.S. Food and Drug Administration (FDA), and have proven to be effective in the treatment of metastatic CRPC (mCRPC) [6,7,8,9,10]. However, many patients treated with these two agents will not experience a PSA response [6,7,8,9], and nearly all of the remaining patients will eventually develop progression despite treatment [11]. Reactivation of the AR is central to the development and pathogenesis of CRPC, and treatment resistance mechanisms may also be mediated by the AR signaling axis. Mechanisms that ultimately alter AR axis signaling, disease progression, and/or lead to treatment resistance in CRPC can be stratified into AR-dependent and AR-independent resistance mechanisms. AR-dependent resistance mechanisms include *AR* amplification, *AR* point mutations, expression of constitutively active *AR* splice variants, and altered intratumoral androgen biosynthesis. AR-independent resistance mechanisms include glucocorticoid receptor overexpression, neuroendocrine differentiation, and immune-mediated resistance mechanisms.

Ultimately, metastatic CRPC (mCRPC) remains incurable, and novel treatment resistance mechanisms continue to be identified, implicating numerous, complex dysregulated molecular signaling pathways that underlie the progression and lethality of the disease. The primary objective of this review article is to discuss the etiologies underlying clinically-relevant mechanisms that lead to drug resistance in mCRPC, and the potential treatment strategies designed to overcome resistance.

## 2. The Human Androgen Receptor

**Figure 1:**
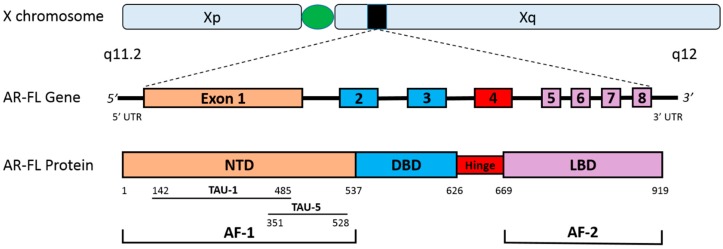
The human androgen receptor gene and protein. This figure depicts the gene and protein structures for the AR-FL. The *AR* is located on the X chromosome (Xq11.2) and is comprised of eight exons. AR-FL contains the NTD (encoded by exon 1), the DBD (encoded by exons 2–3), the hinge region (encoded by exon 4) and the LBD (encoded by exons 5–8). The strong transcriptional activity in the NTD can be attributed to the AF-1, while the LBD contains the weaker AF-2. Two major transactivation units are present in the AF-1: TAU-1 and TAU-5. Abbreviations: AF-1, activation function 1; AF-2, activation function 2; AR-FL, androgen receptor full length; DBD, DNA-binding domain; LBD, ligand-binding domain; NTD, N-terminal transactivation domain; TAU-1, transactivation unit 1; TAU-5, transactivation unit 5; UTR, untranslated region.

Normal differentiation of prostate cells is completely dependent on the AR, and in both androgen-dependent prostate cancer and CRPC, the AR signaling axis plays a central role in disease pathogenesis. The *AR* is a protein coding gene that is located on the X chromosome at Xq11–12, is >90 kb in length, and consists of eight exons. It encodes the human AR protein, which is a member of the steroid hormone receptor superfamily, and a ligand-activated nuclear transcription factor. The AR is 110 kD, comprised of approximately 919 amino acids, and consists of four functional domains: (1) the N-terminal transactivation domain (NTD); (2) the DNA-binding domain (DBD); (3) the hinge region; and (4) the ligand-binding domain (LBD) [12,13,14]. The NTD (amino acids 1–537, encoded by exon 1) is generally considered to be constitutively active, harbors transcriptional activation function-1 (AF-1), and is critical for engaging the cellular transcription complex. Within the AF-1 are two transactivation units (TAU): TAU-1 (amino acids 142–485) and TAU-5 (amino acids 351–528) [15]. Among the two, TAU-5 is responsible for the majority of constitutive transcriptional activity, and has been associated with aberrant AR activation in CRPC cells [16,17]. The DBD (amino acids 538–624, encoded by exons 2 and 3) consists of two zinc finger domains that coordinate AR protein binding to specific DNA sequences, and facilitate receptor homodimerization. The hinge region (amino acids 625–669, encoded by exon 4) separates the DBD from the LBD, and contains the nuclear translocation signal, which is necessary for AR nuclear import. The LBD (amino acids 626–919, encoded by exons 5–8), contains the AF-2, and facilitates binding of androgen ligands, which act as the primary control mechanism of the AR signaling axis (Figure 1) [12,16].

**Figure 2:**
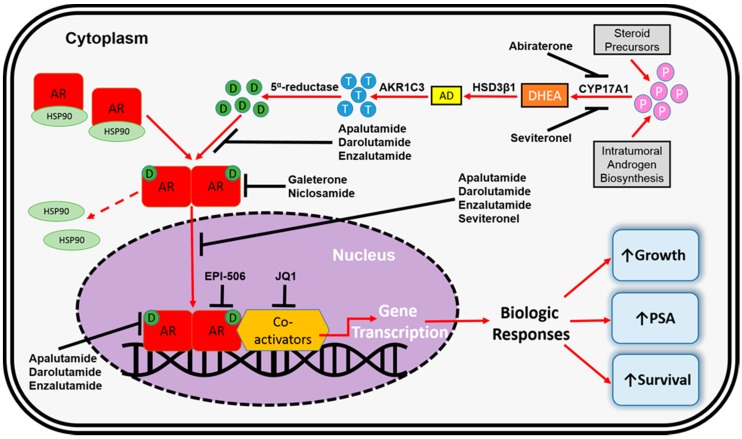
AR signaling axis, and mechanisms of AR targeted inhibition. CYP17A1 is the enzyme responsible for the conversion of androgen precursors (i.e., pregnenolone and progesterone; represented by the light purple circles) to DHEA, while HSD3β1 converts DHEA to AD, AKR1C3 converts AD to testosterone (represented by the blue circles) and finally 5α-reductase converts testosterone to dihydrotestosterone (DHT; represented by the green circles). DHT-mediated activation of the AR causes a conformational change where the AR dimerizes, which then triggers AR translocation into the nucleus. Abiraterone selectively and irreversibly inhibits intratumoral androgen biosynthesis by potently blocking CYP17A1. As a result, less ligand is available for AR activation and AR axis signaling. Seviteronel (VT-464) is also an inhibitor of CYP17A1. Seviteronel has also been shown in preclinical models to have direct inhibitor effects on the AR. Enzalutamide is a potent second-generation antiandrogen that antagonizes the AR, prevents AR translocation into the nucleus, and inhibits AR-mediated transcription. Apalutamide (ARN-509) and darolutamide (ODM-201) are also potent, competitive AR inhibitors with similar mechanisms of action to enzalutamide. EPI-506 reduces AR transcriptional activity by inhibiting protein-protein interactions between the AR and its transcriptional co-regulators. JQ1 is a bromodomain inhibitor that limits AR transcriptional ability by targeting its coactivators. Abbreviations: AD, androstenedione; AKR1C3, aldo-keto reductase family 1 member C3; AR, androgen receptor; CYP17A1, cytochrome P450 c17; DHEA, dehydroepiandrosterone; D, dihydrotestosterone; HSP, heat shock protein; HSD3β1, human 3-beta-hydroxysteroid dehydroxynase/delta5-4 isomerase type 1; P, androgen precursors; PSA, prostate-specific antigen; T, testosterone.

In the absence of dihydrotestosterone (DHT) binding to the AR, it remains isolated in an inactive form within the cytoplasm where it is bound to chaperone proteins (i.e., heat shock protein 90 or HSP90) [18]. In the absence of DHT activation, a nuclear export signal (NES^AR^) helps maintain cytoplasmic localization [19]. However, upon DHT-induced activation of the AR by binding to the LBD, NES^AR^ activity is suppressed, and the AR disassociates from the chaperone complex, undergoes homodimerization, and translocates into the nucleus where it binds to androgen response elements (ARE) in *cis*-regulatory regions of target genes. AR binding to the AREs regulates transcription of genes that elicit biological responses as well as genes responsible for increased growth and survival of the prostate cancer (Figure 2) [18,20]. PSA transcription is predominantly regulated by the AR and therefore, serum PSA levels could be regarded as a surrogate marker of AR activity in tumor cells. When ADT is initiated, reduction in circulating testosterone reduces AR activity in prostate cancer cells and, correspondingly, the serum PSA level decreases.

## 3. FDA-Approved Pharmacotherapeutics for mCRPC

For over seven decades, ADT has been the cornerstone treatment for metastatic prostate cancer, and remains an indispensable treatment paradigm in mCRPC. Although ADT is initially effective in the majority of prostate cancer patients, its effects on tumor growth are transient, and most patients progress within 18–30 months [21]. AR reactivation, manifested by increasing PSA levels or disease progression despite effective testosterone suppression, drives progression to the lethal CRPC phenotype in virtually all patients.

Prior to the FDA approval of abiraterone in 2011 and enzalutamide in 2012, mCRPC patients were traditionally treated with the microtubule-stabilizing taxane, docetaxel. Docetaxel was approved for the treatment of mCRPC based on two seminal phase III clinical trials (TAX 327 and SWOG 9916), both of which demonstrated a modest 2–3-month survival benefit to mCRPC patients [22,23]. After the approval of abiraterone and enzalutamide, docetaxel has largely been relegated to a second- or third-line treatment option for mCRPC. However, the recent ECOG 3805/CHAARTED and STAMPEDE phase III clinical trials demonstrated the effectiveness of docetaxel as a front-line option in patients with metastatic castration-sensitive prostate cancer [24,25].

Abiraterone blocks the production of intratumoral androgen biosynthesis by potently, selectively, and irreversibly inhibiting cytochrome P450 c17 (CYP17A1). CYP17A1 is central to androgen biosynthesis through both 17α-hydroxylase and C17,20-lyase activity [26]. Perhaps most importantly, CYP17A1 is the enzyme responsible for converting pregnenolone to dehydroepiandrosterone (DHEA; Figure 2) [27]. Potent and effective CYP17A1 inhibition ultimately limits the amount of circulating androgens available to activate the AR. Data from two seminal phase III clinical trials (COU-AA-301 and COU-AA-302) led to the FDA approval of abiraterone. The double-blinded, placebo-controlled COU-AA-301 trial was conducted in chemotherapy-pretreated mCRPC patients (*n* = 1195) [6]. COU-AA-301 met its primary endpoint by demonstrating that there was a 3.9-month longer median overall survival (OS) for patients treated with abiraterone (14.8 versus 10.9 months), and a 35% reduction in the risk of death (hazard ratio (HR) = 0.65; 95% confidence interval (CI), 0.54–0.77; *p* < 0.001), when compared to placebo. Patients treated with abiraterone also exhibited significant improvements for all secondary endpoints such as radiographic progression free survival (PFS), time to PSA progression and PSA response rate. COU-AA-302 was a double-blinded, placebo-controlled trial that was conducted in chemotherapy-naive mCRPC patients (*n* = 1088) [7]. COU-AA-302 achieved its co-primary endpoints (OS and radiographic PFS). Investigators observed a 25% reduced risk of death (HR = 0.75; 95% CI, 0.61–0.93; *p* = 0.01), a 47% reduced risk of progression (HR = 0.53; 95% CI, 0.45–0.62; *p* < 0.001), and an increased median PFS by 8.3 months (16.5 versus 8.2 months) in abiraterone-treated patients, when compared to placebo. Again, significant improvements were observed for all secondary endpoints in patients treated with abiraterone. In both trials, excess mineralocorticoid-mediated toxicities were significantly more common among the patients treated with abiraterone. Two recently published clinical trials demonstrated that addition of abiraterone to ADT in metastatic prostate cancer patients who are initiating ADT treatment resulted in substantial benefit, including increased OS and PFS [28,29]. These data may lead to a major shift in standard of care treatment paradigms, where abiraterone is initiated in hormone-sensitive patients. However, as more patients are exposed to abiraterone during earlier stages of disease, complications related to secondary resistance will become even more prominent in clinical practice.

Enzalutamide is a second-generation AR inhibitor that was developed to overcome resistance to first-generation agents (i.e., bicalutamide or flutamide). Enzalutamide has a tri-modal mechanism of action: (1) it potently binds to the AR LBD to prevent ligand binding and AR activation; (2) it inhibits AR translocation into the cell nucleus; and (3) it prevents binding of AR to DNA to effectively inhibit transcription of target genes (Figure 2). One of the main advantages of enzalutamide in mCRPC is that is possesses no agonist properties in mCRPC with *AR* overexpression [30]. Data from two seminal phase III clinical trials (AFFIRM and PREVAIL) led to the FDA approval of enzalutamide. AFFIRM was a double-blinded, placebo-controlled trial conducted chemotherapy-pretreated mCRPC patients (*n* = 1199) [8]. AFFIRM met its primary endpoint by demonstrating that median OS was 4.8 months longer in patients treated with enzalutamide (18.4 versus 13.6 months), and the risk of death was decreased by 37%, when compared to placebo (HR = 0.63; 95% CI, 0.53–0.75; *p* < 0.001). Significant improvements in all secondary endpoints were also observed among patients in the enzalutamide arm. PREVAIL was a double-blinded, placebo-controlled trial conducted in chemotherapy-naıve mCRPC patients (*n* = 1717) [9]. PREVAIL achieved both of its co-primary endpoints (OS and radiographic PFS). Investigators observed a 2.2 month longer median OS (32.4 versus 30.2 months), and a 29% reduced risk of death (HR = 0.71; 95% CI, 0.60–0.84; *p* < 0.001). Second, radiographic PFS was also superior for patients in the enzalutamide arm, with an 81% reduced risk of progression (HR = 0.19; 95% CI, 0.15–0.23; *p* < 0.001). Similar to AFFIRM, patients in the PREVAIL enzalutamide arm also achieved significant improvements in all secondary endpoints.

The pharmacotherapeutic landscape for the treatment of mCRPC has been further expanded over the last decade with the FDA approval of three new agents. Options for the treatment of mCRPC now include: (1) the autologous cellular immunotherapy, sipuleucel-T, for asymptomatic or minimally symptomatic mCRPC patients (approved by the FDA in 2010) [31]; (2) the semi-synthetic taxane, cabazitaxel, which was shown to be effective in overcoming secondary resistance to docetaxel (approved by the FDA in 2010) [32]; and (3) the α-emitting radiopharmaceutical, radium-223 (approved by the FDA in 2013) [33]. However, resistance mechanisms for these agents will not be discussed, as they are beyond the focus of this review. Despite the availability of enzalutamide and abiraterone for CRPC patients, secondary resistance mechanisms inevitably result in clinical progression. Novel therapeutics that target altered intratumoral androgen biosynthesis (i.e., seviteronel [34]) or the AR with greater affinity and potency (i.e., apalutamide and darolutamide [35,36]), as well as those that degrade the AR (i.e., niclosamide [37]) or target the AR NTD (i.e., EPI-506 [38,39]) are currently in development for the treatment mCRPC (Figure 2). These currently investigational pharmacotherapeutics will be discussed in more detail in the following sections.

## 4. AR-Dependent Resistance Mechanisms

Although abiraterone and enzalutamide have advanced the treatment of mCRPC patients, approximately 20–40% of patients present with primary resistance to these agents (e.g., no initial PSA response) [6,7,8,40]. Moreover, patients who do experience a clinical or biochemical response after treatment with these two agents will eventually develop secondary resistance to the drug [11]. Despite distinct mechanisms of drug action, there may be significant cross-resistance between abiraterone and enzalutamide [41,42]. One plausible hypothesis of cross-resistance between the two drugs centers on the recent finding that an active metabolite of abiraterone (Δ4-abiraterone) has potent AR antagonist properties. The mechanisms of drug action that are similar between Δ4-abiraterone and enzalutamide could also be shared resistance mechanisms that explain the cross-resistance between the two drugs [43].

Primary and secondary resistance to abiraterone and enzalutamide are extremely complex [44]. Mechanisms of resistance that are mediated by the AR include (but are likely not limited to): *AR* amplification, *AR* overexpression, *AR* somatic point mutations, constitutively active *AR* splice variants, and altered intratumoral androgen biosynthesis [18,45,46,47].

### 4.1. AR Amplification and Overexpression

*AR* amplification and overexpression are two primary etiologies for progression to the mCRPC phenotype, and are likely important to the development of treatment resistance. While not generally present in hormone-sensitive cells, up to 80% of CRPC cells exhibit *AR* amplification, *AR* mRNA overexpression, or AR protein overexpression [48,49]. *AR* amplification, leading to AR overexpression, enables progression to CRPC even in the setting of low circulating androgens due to ADT treatment [50].

In addition to its role in mCRPC progression, there is mounting evidence that *AR* amplification could also be an important resistance mechanism. Preclinical in vitro studies using enzalutamide-resistant LNCaP cells express higher levels of AR (and AR splice variants) when compared to naïve LNCaP cells [51]. One in vivo study showed 3-fold increased AR expression after CRPC xenografts were treated with abiraterone [52]. Finally, a recent study that utilized liquid biopsies and circulating tumor DNA (ctDNA) to probe the *AR* genomic landscape discovered that patients with *AR* amplification were less likely to respond to treatment. A total of 50% of patients who were pretreated with either enzalutamide or orteronel (a CYP17A1 inhibitor) prior to abiraterone treatment showed evidence of *AR* amplification, and only 13% of those with *AR* gain demonstrated a response with ≥50% PSA decline after being treated with abiraterone [53]. A separate study, using circulating tumor cells (CTCs), examined *AR* amplification as a resistance mechanism in mCRPC patients treated with abiraterone or enzalutamide, and who had received previous docetaxel treatment. Among the patients in the study discovery cohort who had received docetaxel (*n* = 98), *AR* amplification was associated with a worse rate of PSA decline of ≥50%, shorter PFS (HR = 1.95; 95% CI, 1.23–3.11; *p* < 0.01), and shorter OS (HR = 3.81; 95% CI, 2.28–6.37; *p* < 0.001). These results were confirmed in their replication cohort of enzalutamide-treated patients from the PREMIERE trial (*n* = 100), where patients with *AR* amplification experienced shorter PSA PFS (HR = 4.33; 95% CI, 1.94–9.68; *p* < 0.001), and OS (HR = 11.08; 95% CI, 2.16–56.95; *p* < 0.004) [54]. Additional studies of liquid biopsies have also demonstrated that *AR* amplification in ctDNA is associated with resistance to abiraterone and enzalutamide [55,56].

### 4.2. AR Point Mutations

**Figure 3:**
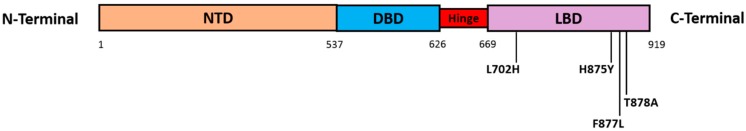
AR somatic missense mutations. The main four missense mutations that are focused on in this review all occur in the AR LBD (*AR* exons 5–8) and include: L702H, H875Y, F877L (previously published as F876L), and T878A (previously published as T877A). Abbreviations: DBD, DNA binding domain; LBD, ligand binding domain; NTD, N-terminal transactivation domain.

In CRPC, AR mutations are found in 5–30% in tumors, CTCs, and ctDNA [53,55,57]. *AR* point mutations confer resistance to enzalutamide and abiraterone, but currently there is some ambiguity as to whether the spectrum of somatic mutations that confer drug resistance are different for abiraterone and enzalutamide. The majority of clinically-relevant somatic point mutations in the *AR* is located in the LBD. These include four main somatic missense mutations: (1) a leucine to histidine substitution at amino acid 702 (L702H); (2) a histidine to tyrosine substitution at amino acid 875 (H875Y); (3) a phenylalanine to leucine substitution at amino acid 877 (F877L); and (4) a threonine to alanine substitution at amino acid 878 (T878A) (Figure 3).

One of the most frequently reported *AR* point mutations is T878A (previously reported in the literature as T877A), which is a gain of function mutation, and can be activated by both steroid hormones (e.g., progesterone) and first-generation antiandrogens (e.g., bicalutamide or flutamide). It most commonly arises after treatment with abiraterone because when CYP17A1 is effectively inhibited, intracellular progesterone levels increase, while DHEA and testosterone are suppressed. Intuitively, it would appear that this mutation would effectively limit AR activation; however, the T878A mutation broadens ligand binding specificity of the AR so that it can be activated by progesterone, glucocorticoids, and estrogen [58]. Thus, the T878A mutation creates malignant clones that are able to overcome abiraterone inhibition [59]. A recent study of abiraterone-treated mCRPC patients revealed that the T878A mutation was detected in 3 of 18 patients at high frequency [60].

A second frequently reported somatic mutation, F877L (previously reported in the literature as F876L), arises in patients after treatment with enzalutamide or apalutamide. Apalutamide (formerly ARN-509) and darolutamide (formerly ODM-201) have a similar mechanism of action to enzalutamide [35,36]. There is evidence that darolutamide could more potently antagonize the AR than enzalutamide [36]. In preclinical models, both have shown less blood–brain barrier penetration than enzalutamide, which could therefore spare patients the central nervous system-mediated adverse events (i.e., seizures) associated with enzalutamide [35,36]. Preclinical data from prostate cancer cell lines and patient tumor tissues revealed that F877L can effectively convert enzalutamide from a potent antagonist into a partial agonist [61,62,63]. Another preclinical study demonstrated that F877L can occur spontaneously in enzalutamide-treated cells, suggesting that this could be an important secondary resistance mechanism for second-generation AR antagonists [62]. Spontaneous F877L mutations were also detected in ctDNA of patients treated with apalutamide or enzalutamide [55,61]. However, darolutamide has been shown, preclinically, to be resistant to both T878A and F877L point mutations [36], which might make it an attractive option for patients who develop secondary resistance to enzalutamide. Additional preclinical data has revealed that the nonsteroidal CYP17 inhibitor seviteronel (formerly VT-464) cannot only potently antagonize the AR, but it can also overcome F877L mutations that arise after treatment with enzalutamide or abiraterone [34,64,65].

Interestingly, L702H is a mutation that can result in glucocorticoid-mediated activation of the AR. In one study, the L702H mutation was associated with primary resistance to abiraterone [66]. Another study demonstrated that both L702H and T878A point mutations were associated with poor PSA response after abiraterone or enzalutamide treatment. Among patients with aberrant *AR* who were treated with enzalutamide, only 13% experienced a ≥50% PSA decline after abiraterone treatment [53]. Another recent study demonstrated that abiraterone-treated patients harboring either the T878A or the L702H mutation experienced shorter OS (HR = 3.26; 95% CI, 1.47–not reached; *p* < 0.004), when compared to patients without detectable point mutations. In this study, T878A and L702H mutations were only found in patients who had received prior docetaxel treatment [54].

### 4.3. AR Splice Variants

**Figure 4:**
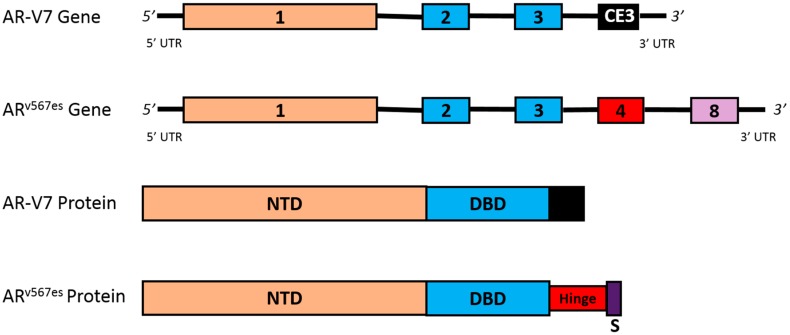
Clinically-relevant splice variants. This figure depicts the gene and protein structures for the AR-V7 and AR^v567es^ splice variants. Alternate splicing of the *AR* leads to the formation of the constitutively active, and clinically-relevant, AR-V7 and AR^v567es^ splice variants. AR-V7 is a variant with a cryptic exon 3 instead of exons 4–8. This alternative splicing leads to a protein that has lost the hinge region and LBD. AR^v567es^ is a variant that contains full sequences of exons 1–4, and exon 8; however, exons 5–7 are skipped. As a result of alternative splicing, a frameshift causes the creation of a premature stop codon in exon 8. Both splice variants are constitutively active proteins that can bind to DNA to promote transcription without the need for ligand binding. Abbreviations: AR^v567es^, androgen receptor splice variant with exons 5–7 skipped; AR-V7, androgen receptor splice variant V7; CE3, cryptic exon 3; NTD, N-terminal transactivation domain; S, premature stop codon; UTR, untranslated region.

Approximately 20 AR mRNA splice variants (AR-V) have been identified since 2008. While select AR-Vs are only conditionally active (i.e., AR-V1 and AR-V9) [67], many are constitutively active (i.e., androgen-independent nuclear localization to promote transcription of target genes) [68], and are likely to be a clinically-relevant mechanism of secondary resistance in mCRPC patience who have been treated with abiraterone or enzalutamide. While most AR-Vs retain the NTD and DBD domains, alternative splicing of AR-V7 results in the addition of a cryptic exon 3. In AR^v567es^, alternate splicing leads to skipping of exon 5, 6, and 7, and a frameshift that causes a premature stop codon in exon 8. Both splice variants cause the loss of the LBD domain, and the formation of the C-terminal truncated protein (Figure 4) [69,70,71].

AR-V expression has been associated with both enzalutamide and abiraterone resistance. In preclinical xenograft models, it was shown that expression of AR-V7 and AR^v567es^ can be induced by abiraterone. In one study that demonstrated an OS advantage in mice treated with abiraterone, AR-V7 and AR^v567es^ expression was increased 3.1-fold and 5.2-fold, respectively [52]. Similarly, increased splice variant mRNA expression was discovered in mouse xenograft models of enzalutamide-resistance [52,72].

Clinically, AR^v567es^ and AR-V7 are likely to be the active in mCRPC, and most relevant to enzalutamide and abiraterone resistance. Early studies revealed that expression levels of AR^v567es^ and AR-V7 were correlated with poorer survival in patients [73]. Moreover, AR-V7 may be a predictive biomarker of secondary resistance and poor outcomes in mCRPC that will inform treatment selection, and aid in future development of therapeutics. A recent study (*n* = 62), utilizing a CTC assay, demonstrated that AR-V7 mRNA expression is associated with enzalutamide and abiraterone secondary resistance [11]. In this study, CTCs from 31% of the enzalutamide-treated patients and 19% of the abiraterone-treated patients demonstrated detectable AR-V7 mRNA expression. Patients who were treated with either enzalutamide or abiraterone, and were positive for AR-V7, achieved significantly lower rates of PSA response, and experienced significantly shorter PFS and OS. This observation could also provide an additional mechanism of cross-resistance between these two agents. Interestingly, AR-V7 mRNA expression can increase from baseline in patients treated with either taxanes, abiraterone, or enzalutamide, but a decline in AR-V7 levels was only observed in patients treated with a taxane [74]. Additionally, a separate study that used CTCs to detect AR-V7 mRNA revealed that patients with detectable AR-V7 may benefit more from taxane treatment, when compared to either abiraterone or enzalutamide treatment [75]. These data suggest that patients maintain sensitivity to taxanes, despite the presence of detectable AR-V7 mRNA, which means that taxanes remain a viable treatment option for patients who progress on abiraterone and/or enzalutamide.

All of the current FDA-approved medications target the LBD, but it is the NTD that contains the transcriptionally active portion of the AR (AF1). Because clinically-relevant AR-Vs, such as AR^v567es^ and AR-V7, confer resistance, compounds that degrade the AR (i.e., niclosamide [37,76], or even galeterone [77] as a proof of concept), prevent AR-mediated transcription (i.e., bromodomain-containing protein 4 (BRD4) inhibitors like JQ1 [78]), or target the AR NTD (i.e., sintokamides [79] or EPI-506 [38,39]) could be viable treatment options after patients progress on abiraterone and/or enzalutamide.

Niclosamide is an FDA-approved anti-helminthic drug that was shown in preclinical models to be particularly effective at targeting AR-V7 through proteasome-dependent downregulation of AR-V7 protein expression, and inhibition of AR-V7 transcriptional activity through reduced AR-V7 recruitment to the *PSA* promoter. In addition, the combination of niclosamide and enzalutamide was shown to inhibit tumor growth in enzalutamide-resistant in vitro models, indicating that niclosamide may be a viable option to overcome secondary resistance to enzalutamide in mCRPC [37]. Moreover, the combination of niclosamide and abiraterone was also shown to resensitize abiraterone-resistant AR-V7 cells in both in vitro and in vivo preclinical models of CRPC [76].

EPI-506 is a prodrug of one of the four stereoisomers of its predecessor, EPI-001. EPI-506 binds to the AF1 region, and thereby effectively blocks the NTD, inhibits AR transcriptional activity by reducing protein–protein interactions between the AR and co-activators, and prevents NTD transactivation [38,39]. This mechanism conceivably allows EPI-506 to overcome the common secondary resistance mechanisms germane to agents that target the LBD. Preclinically, analogs of EPI-001 were able to inhibit transcription of cells with full-length AR, as well as those with the AR^v567es^ splice variant. These analogs of EPI-001 inhibited the growth of xenograft tumors that express AR splice variants lacking the LBD [39]. Currently, a phase I/II study with EPI-506 is underway to evaluate the safety and efficacy or EPI-506 in treatment-naïve mCRPC patients, and also those who have progressed on enzalutamide or abiraterone (NCT02606123). Correlative studies using CTCs will assess the efficacy of EPI-506 in patients with detectable levels of AR-V7 mRNA.

An alternative approach to bypass secondary resistance mechanisms involving AR splice variants is to target co-activators or co-repressors involved in AR-mediated transcription. Numerous molecules that act as either co-activators or co-repressors of the AR, to modulate its transcriptional activity, have been identified [80]. Specifically, the AR co-activator BRD4 is a potential pharmacotherapeutic target in mCRPC. BRD4 recruits RNA polymerase II (RNA PolII) and the transcription elongation factor P-TEFb to promote transcription. JQ1 competitively binds to BRD4, displaces it from active chromatin, and removes RNA PolII from target genes [81]. One preclinical study showed that JQ1 prevented BRD4 binding to the AR NTD, and thus mediated inhibition of AR binding to chromatin enhancer sites. But more importantly, this study demonstrated that JQ1 effectively inhibited AR-V7 and AR^v567es^ mRNA and protein expression [82]. Then, in a second preclinical study, a panel of prostate cancer cell lines were shown to be sensitive to JQ1-mediated cell cycle arrest and apoptosis. Treatment of enzalutamide-resistant VCaP cells resulted in transcriptional downregulation of AR target genes. Perhaps most importantly, in mouse xenograft models of treatment resistance where elevated levels of AR-V7 were detected, JQ1 monotherapy (or in combination with enzalutamide) effectively delayed tumor progression, and showed robust silencing of AR-V7 [81]. These data point to the potential of bromodomain inhibitors to overcome secondary treatment resistance mechanisms mediated by expression of AR splice variants.

### 4.4. Altered Steroidogenesis

It is likely that extended treatment with either abiraterone or enzalutamide induces alterations to intratumoral androgen biosynthesis. As a result, increased circulating androgens, combined with mutations (germline and/or somatic) that affect metabolizing enzyme expression or function, promote AR reactivation and progression to mCRPC. For instance, enzalutamide-resistant cell lines were found to have upregulated androgens, and over-expressed genes involved in androgen biosynthesis [83]. Multiple gene expression studies have identified significantly increased levels of *SRD5A1*, *HSD3β1*, and *AKR1C3* in CRPC tissues [84,85,86]. Enzalutamide-resistant xenograft tumors revealed increased protein aldo-keto reductase family 1 member C3 (AKR1C3) protein expression [83]. Abiraterone-treated xenografts with the gain-of-function N367T missense mutation (asparagine to threonine substitution at amino acid 367) in *HSD3B1* were resistant to ubiquitination and degradation, which led to DHT accumulation [87]. Finally, abiraterone-treated tumor xenografts revealed 2-fold upregulation of CYP17A1 [52], and cell-free *CYP17A1* copy number variations were associated with poorer outcomes in abiraterone-treated patients [88].

## 5. AR-Independent Resistance Mechanisms

In addition to AR-mediated resistance mechanisms, there are several AR-independent resistance mechanisms that lead to treatment failure, as well as progression of mCRPC. Glucocorticoid receptor overexpression, neuroendocrine differentiation, and immune-mediated resistance have also been implicated in treatment resistance in CRPC [58,89,90].

### 5.1. Glucocorticoid Receptor Activation

The role of glucocorticoids and the glucocorticoid receptor (GR) in prostate cancer is complex because glucocorticoids can be both beneficial and harmful. However, in mCRPC where ADT is used to antagonize AR signaling, GR upregulation and activation can be another clinically-relevant mechanism of resistance to therapeutics that target the AR signaling axis.

The AR and GR are both members of the same class of nuclear steroid receptors, share common structures and mechanisms of action [58], have highly homologous DBDs [91], and have overlapping transcriptomes [92].

As patients progress to mCRPC, the reliance on direct AR signaling is bypassed as a result of potent receptor inhibition, which causes subsequent activation of the GR. Activated GR is then able to bind to nuclear AREs and regulate a subset of AR target genes that promote cell survival and tumor progression [92,93]. One recent preclinical study provided evidence showing GR-driven resistance in two independent in vitro models (LNCaP/AR and VCaP cells). This study also showed increased GR expression was associated with both enzalutamide and apalutamide resistance. In addition, the authors also demonstrated that GR knockout in VCaP cells could restore sensitivity to enzalutamide [92]. A separate preclinical study using chromatin immunoprecipitation (ChIP) combined with deep DNA sequencing revealed that GR protein expression is negatively controlled by AR signaling. In addition, xenograft models from this study also showed that *GR* mRNA and GR protein expression increased in the presence of *AR* knockdown or AR antagonists [91]. Moreover, in vitro models have also revealed that GR overexpression was associated with docetaxel resistance, and that GR antagonism could resensitize docetaxel-resistant prostate cancer cells [94].

Data from these publications support the hypothesis that GR activation and upregulation, secondary to AR antagonism, is a potential resistance mechanism to therapies targeting the AR axis. Further investigations with analyses of pre-treatment patient samples (i.e., blood), as well as samples at the point of treatment progression, are necessary to characterize the extent to which GR activation and upregulation contributes to secondary resistance in mCRPC.

### 5.2. Immune-Mediated Resistance Mechanisms

Despite FDA approval of several immunotherapies for a variety of malignancies within the past three years, the role of checkpoint inhibitors for the treatment of mCRPC has yet to be fully elucidated. Primarily, this was based on negative results from studies involving the CTLA-4 inhibitor, ipilimumab [95]. However, a recent analysis of PD-L1 expression was performed in two independent, well-characterized prostate cancer cohorts. In the discovery cohort (*n* = 209), moderate to high PD-L1 expression in prostatectomy specimens was associated with tumor progression (Ki-67, *p* < 0.001), Gleason score (*p* = 0.004), AR expression (*p* < 0.001), and was prognostic for biochemical recurrence (HR = 2.37; 95% CI, 1.32–4.25; *p* = 0.004). In the replication cohort (*n* = 611), associations between PD-L1 and AR expression, and proliferation were confirmed (*p* < 0.001). Furthermore, the association between PD-L1 expression and biochemical recurrence was confirmed in a multivariate model (HR = 1.46; 95% CI, 1.11–1.92; *p* = 0.007) [96]. Recent preclinical in vitro and in vivo studies have revealed that PD-L1 is significantly expressed in enzalutamide-resistant cell lines, and that mice with enzalutamide-resistant tumors also experienced detectable circulating PD-L1 levels [97]. Results from these studies support the hypothesis that mCRPC progression and resistance to AR signaling axis inhibitors may be mediated by PD-L1 and PD-1. To further support this hypothesis, a phase II clinical trial is underway to investigate the use of the PD-1 inhibitor pembrolizumab (in combination with enzalutamide) in mCRPC patients who developed secondary resistance to enzalutamide (NCT02312557). Initial results from the first 10 patients enrolled on the trial have recently been published, and showed that three of these patients experienced rapid PSA reductions (≤0.2 ng/mL), and two of the three achieved a partial response [98].

There are two additional ongoing phase II trials exploring the role of immunotherapy in mCRPC. One of the two trials is evaluating ipilimumab in combination with abiraterone in treatment-naïve mCRPC patients (NCT01848067), and could conceivably answer important hypotheses pertaining to primary resistance. More importantly, the second trial is evaluating combination immune checkpoint blockade with ipilimumab and the PD-1 inhibitor nivolumab in mCRPC patients positive for AR-V7 (NCT02601014). This second trial has the potential to be informative from the perspective of using a predictive biomarker for the purpose of treatment selection, and also for understanding the role of immunotherapy in pre-treated mCRPC patients.

### 5.3. Neuroendocrine Differentiation

While only approximately 1% of all primary prostate cancers are diagnosed as neuroendocrine prostate cancer (NEPC), up to 30% of mCRPCs are NEPC [99]. The progression of prostate cancer adenocarcinoma (PCA) to NEPC has become increasingly appreciated recently as an important mechanism of treatment resistance, which ultimately results in transition to an extremely lethal prostate cancer subtype. This profound phenotypic switch, as a consequence of selective pressure from ADT or potent antiandrogens, from tumors with adenocarcinoma histologic features that express AR to AR-negative neuroendocrine prostate tumors, has been termed lineage plasticity. This situation is somewhat analogous to the emergence of small cell lung cancer in epidermal growth factor receptor-mutant adenocarcinoma treated with the inhibitor of epidermal growth factor receptor [100]. In a study of metastatic biopsies from 81 prostate cancer patients (*n* = 51 PCA, and *n* = 30 NEPC), whole exome sequencing showed that molecular signatures and gene copy numbers were similar between the two prostate cancer subtypes, confirming that NEPC is derived from PCA precursors [101]. Until recently, selection of appropriate and effective treatments for NEPC has been thwarted by a poor understanding of molecular drivers of lineage plasticity in prostate cancer. 

Loss or mutation of both *TP53*, which encodes the p53 tumor suppressor protein, and *RB1*, which encodes the retinoblastoma tumor suppressor protein, have emerged as two important factors in NEPC differentiation [102,103]. In one study, RB1protein loss was observed in 90% of NEPC tumors, and *RB1* deletions in 85% of cases [104]. A recent preclinical in vivo mouse study revealed that *Rb1* loss facilitates lineage plasticity and metastasis in PCA with *Pten* loss, and *Trp53* causes secondary resistance to therapies targeting the AR signaling axis [105]. A second preclinical study, using in vitro and in vivo human prostate cancer models, demonstrated evidence of lineage plasticity, and a shift from androgen-dependent PCA to androgen-independent NEPC after treatment with enzalutamide. This phenotypic switch was facilitated by loss of *TP53* and *RB1*, and was mediated by increased expression of a transcription factor named *SOX2*. They also showed that inhibition of *SOX2* restored *TP53* and *RB1* function [106]. 

Genomic amplification and overexpression of *MYCN*, which encodes N-Myc, and *AURKA*, which encodes Aurora kinase A, are also associated with differentiation from PCA to NEPC [104,107,108]. Two studies showed that amplifications of both *MYCN* and *AURKA* occurred in between 40–86% of pretreated NEPC samples and metastases, compared to only 5% of PCAs [107,108]. Another recent study showed that PCA and NEPC arise from a common epithelial clone, that N-Myc is an NEPC driver, and that inhibition of Aurora kinase A destabilizes N-Myc [109]. In another preclinical study, N-Myc abrogated AR signaling, and N-Myc protein overexpression drove an aggressive cancer that is molecularly similar to human NEPC. In this study, Aurora kinase A knockdown, or treatment with the Aurora kinase A inhibitor alisertib (formerly MLN8237) resulted in decreased N-Myc protein levels, N-Myc target gene expression, and cell viability [110]. 

There are currently no therapeutics in the drug development pipeline that target *TP53* or *RB1* genomic loss or mutations. However, a phase II trial is underway, which is evaluating the safety and effectiveness of alisertib in mCRPC and NEPC patients (NCT01799278). A second Aurora kinase A inhibitor, CD532, was shown to reduce N-Myc protein levels in preclinical models driven by *MYCN*, which indicates this could conceivably be a viable treatment option for NEPC patients in the future if the drug is moved into clinical trials [109].

## 6. Conclusions

Nearly all patients with metastatic prostate cancer who are initially treated with ADT will progress to mCRPC, mainly due to reactivation of the AR signaling axis. Because of myriad adaptive resistance mechanisms, mCRPC remains incurable despite the development and FDA approval of novel agents that target the AR signaling axis. However, the identification and a more comprehensive understanding of AR-dependent (i.e., *AR* amplification, *AR* point mutations, expression of constitutively active *AR* splice variants, and altered intratumoral androgen biosynthesis), and AR-independent mechanisms (i.e., glucocorticoid receptor activation and upregulation, neuroendocrine differentiation, and immune-mediated resistance) will allow for the design and implementation of novel pharmacotherapeutic treatment paradigms in patients with mCRPC. Moreover, the development, validation and clinical implementation of assays (i.e., liquid biopsies) will be useful in identifying mechanisms of resistance, can conceivably provide clinically relevant predictive biomarkers, and will become essential tools that help clinicians identify the optimal treatment for a given patient.
